# Bidirectional temporal relationships between emotional state and eating across eating disorders: a network approach

**DOI:** 10.1186/s40337-026-01617-7

**Published:** 2026-04-26

**Authors:** Sercan Kahveci, Julia Reichenberger, Ann-Kathrin Arend, Alessandra C. Mansueto, René Freichel, Ulrich Voderholzer, Jens Blechert

**Affiliations:** 1 Department of Psychology and Centre for Cognitive Neuroscience, Paris-Lodron-University of Salzburg, Hellbrunner Straße 34, 5020 Salzburg, Austria; 2 Department of Psychology, Ludwig Maximilian University Munich, Leopoldstraße 13, 80802 Munich, Germany; 3 Centre for Urban Mental Health, University of Amsterdam, Oude Turfmarkt 145-147, 1012GC Amsterdam, The Netherlands; 4 Department of Communication Science, University of Amsterdam, Nieuwe Achtergracht 166, 1018WV Amsterdam, The Netherlands; 5 Department of Psychology, University of Amsterdam, Nieuwe Achtergracht 129-B, 1018WS Amsterdam, The Netherlands; 6 Department of Psychiatry and Psychotherapy, University Hospital of the LMU Munich, Nußbaumstraße 7, 80336 Munich, Germany; 7 Schön Clinic Roseneck, Am Roseneck 6, 83209 Prien am Chiemsee, Germany

**Keywords:** Eating disorders, Symptom networks, Time-series, Dynamics, Ecological momentary assessment

## Abstract

**Objective:**

Several models of eating disorders (EDs) suggest that emotions and eating influence each other in a vicious cycle, producing part of the observed symptoms. However, no research tested whether such cycles exist across EDs. We therefore explored networks of prospective relationships between negative and positive emotions and eating-related behaviors (hunger, food craving, calorie intake, and binges) across EDs and healthy controls (HCs).

**Method:**

These variables were assessed six times a day for eight days in women with restrictive (AN-R, *N* = 29) and binge-purge (AN-BP, *N* = 26) Anorexia Nervosa, Bulimia Nervosa (BN, *N* = 42), and Binge-Eating Disorder (BED, *N* = 37), and in HCs (*N* = 57). Prospective relationships were analyzed with modified vector-autoregressive networks.

**Results:**

Unlike HCs, ED groups showed many emotion-eating relationships. Calorie intake predicted subsequent increased negative or reduced positive affect in every ED, and so did binges in AN-BP, BN, and BED; post-binge negative affect was predicted to last up to 10 h. AN-R showed positive emotional eating, with more desire to eat and hunger during positive mood, while BED showed negative emotional eating, with worry predicting desire to eat and hunger, and irritation predicting calorie intake directly. Binges were not predicted by negative emotions, likely due to a long temporal distance between measurements. All EDs showed restriction-promoting feedback loops mediated by negative affect following calorie intake. In particular, AN-BP showed a panic-like cycle wherein a lack of hunger predicted worry, and this worry predicted a further reduction in hunger.

**Discussion:**

Post-eating dysphoria was present in all EDs, while restriction-promoting feedback loops were present in most EDs.

## Abstract (plain language summary)

This study investigated the relationship between emotions and eating, hunger, and desire to eat in people with eating disorders (EDs). The study focused on individuals diagnosed with four different eating disorder subtypes: restrictive anorexia nervosa (AN-R, 29 women), binge-purge anorexia nervosa (AN-BP, 26 women), bulimia nervosa (BN, 42 women), and binge-eating disorder (BED, 37 women); additionally, healthy participants were included for comparison (HCs, 57 women). These individuals used a mobile phone app 6 times per day for 8 days to report how they feel, what they ate, how hungry they were, and how strongly they wanted to eat. Results suggest that individuals with all EDs felt worse emotionally after a meal, unlike HCs. Individuals with AN-BP, BN, and BED also reported feeling more negative after a binge eating episode, contrary to the idea that binge eating helps people feel better. Individuals with AN-R seemed more willing to eat after they felt happy. In contrast, individuals with BED were more willing to eat after they felt worried, and actually ate more after feeling irritated. Individuals with EDs were also less willing to eat after other specific negative emotions. They thus seemed to experience a vicious cycle in which a meal is followed by negative feelings, which are, in turn, followed by less willingness to eat more. This confirms pre-existing theories on why and when individuals with EDs are not willing to eat.

## Introduction

Eating disorders (EDs) are associated with severe physical complications like cardiovascular disease and diabetes mellitus, as well as psychological comorbidities like depression, anxiety, and substance abuse disorders [62], e.g., [8]. The DSM-5 [3] distinguishes three main EDs. Of these, Anorexia Nervosa (AN) is characterized by restrained eating and, typically, underweight, and consists of two subtypes: the binge-purge subtype (AN-BP) is characterized by binge-eating episodes or compensatory behavior, and the restrictive subtype (AN-R) lacks these characteristics; Bulimia Nervosa (BN) and Binge-Eating Disorder (BED) are marked by frequent binge-eating episodes, respectively with and without compensatory behavior. To tailor interventions for eating disorders, it is important to understand what drives binge-eating, overeating, and restricted eating.

### Negative affect triggers binge eating

Individuals often eat more than intended when experiencing specific emotions, a phenomenon known as *emotional eating*. Models of binge eating often explain the behavior as resulting from negative affect [16]. This is indeed observed in naturalistic research in ED samples [29] and retrospectively reported by patients with BED and BN [54], with negative affect being the most commonly reported antecedent of binge eating [68]. Other models go one step further and propose that binge eating serves to *reduce* negative affect when it occurs [40, 45]. This has been supported by studies which analyze mood trajectories leading up to the binge and following the binge across the day (e.g., [10, 56, 69]); these studies draw a picture of negative affect gradually rising up to the moment a binge occurs, after which negative affect gradually decreases. The idea that binges relieve negative affect is contradicted by studies that test differences in affect directly before and after a binge however: these studies find that negative affect *increases* after a binge [2, 29, 34, 66], or that some negative emotions are soothed while others are worsened or remain unchanged [13, 63].

### Eating as an indirect trigger for restriction in anorexia and bulimia nervosa

Several models suggest that *restriction* likewise fulfills an emotion regulatory role in AN and BN: one model suggests that restriction temporarily flattens mood, while eating brings about physical and emotional distress due to physiological changes and an intolerance of mistakes [58]; others suggest that restriction gives individuals a sense of control which they may otherwise lack [26] or soothes fears of weight gain [3]. These models hence imply a negative feedback loop: perceived failures in restriction take away the individual’s sense of self-control or otherwise bring about distress, which is repaired with an intensification in restriction. Critically then, the path from perceived overeating to restriction is mediated by negative affect. Do we see this in daily life?

In AN, it is indeed found that eating is followed by negative affect [4, 27], and negative affect promotes eating restriction [22, 32], while restriction is followed by positive affect and a reduction in guilt [31]. In BN, it is found that negative affect increases after a binge as discussed above, but also after a normal meal (though another study does not find this: [4, 34]), and that pathological restriction increases after self-criticism (in a predominantly BN sample: [47]). In both AN and BN, negative affect also predicted meal-skipping, and subsequently, meal-skipping predicted positive affect [12]. Experimentally induced positive affect was also found to buffer against different parts of this cycle in both groups: individuals with AN ate more from a test meal, and individuals with BN experienced less anxiety from eating [17]. Hence, each step in this hypothetical negative feedback loop finds support in the literature. What is so far lacking, however, is a study that tests for the presence of the complete cycle in the same sample. Psychological network modeling may be a suitable means of testing this.

### Characteristics of network models

*Dynamic network modeling approaches* can model the full range of bidirectional associations of emotions and eating over time, and thus reveal vicious cycles. They visualize these associations in network graphs depicting the variables as ‘nodes’, and the relationships between them as ‘edges’. Network analysis has become prominent in psychopathology in general and EDs in particular (for reviews see [43, 49, 60, 64]) as it can identify targets for intervention. Particularly suited to our research question is the multilevel vector autoregressive (VAR) network model. This method models how deviations in each variable can predict deviations in all variables at the next timepoint. This reveals *prospective relationships* between variables, while controlling for (i.e., partialing out) effects held in common by multiple variables. The resulting network can reveal how different nodes and edges form feedback loops; through these loops, deviations in certain variables may be prolonged (positive feedback loops) or suppressed (negative feedback loops). Likewise, the network can reveal how perturbations in one variable may spread through the network and influence other variables over time. Beyond visual inspection of the network, this shows how strong and how long a perturbation in one variable would affect other variables.

### Past network modelling research on emotions and eating

So far, there has been no network-based research studying the *temporal* interplay of emotions and eating in separate EDs [49]; most studies incorporating a temporal aspect have investigated negative cognitions more broadly, left regular eating out of the picture, and studied mixed ED samples. One study used EMA to create temporal networks for individual patients with EDs to identify personalized central therapeutic targets [42]. This study found high variability in which nodes were most central, but negative emotions and cognitions represented 8 of the 10 most and second-most common central nodes. Another study in individuals with EDs found that momentary fear of weight gain predicts restriction at the next timepoint, but restriction was further predicted by thoughts about dieting and feeling like one does not deserve to eat, as well as other behaviors such as body checking, compensation, and exercise [41]. Finally, one study in a mixed ED sample found that subjective overeating was preceded by guilt and a desire to be thinner, while binges were not preceded by any negative cognitions; this study also found that binges led to guilt, an emotion which did not directly predict any overeating behavior; perceived overeating, meanwhile, predicted reductions in desire to be thinner or to diet, altogether contradicting the idea that overeating and binges result in compensatory behavior [39].

Besides these studies, specific relationships between emotions and eating behavior were also revealed by the few cross-sectional network analysis studies looking into emotion in ED samples: in patients with AN and BN, different symptoms were connected to each other by guilt after overeating [57, 61, 70] and fear of weight gain [28, 44]. In contrast, eating and affective features were less central in a BED sample, compared to self-monitoring metacognition and impulse control [1].

### Aims of this study

The central goal of our study was to investigate the daily temporal relationships between emotions and eating behavior in different EDs. Hence, we used smartphone-based ecological momentary assessment (EMA) to ask interview-diagnosed individuals with EDs (AN-R, AN-BP, BN and BED) and healthy controls (HCs) about their emotions and eating behavior six times per day for eight days. Among eating-related behaviors and states, we assessed calorie intake, desire to eat, hunger, and the occurrence of binges. After estimating disorder-specific temporal networks from this data using multilevel vector-autoregression (VAR) models, we performed several analyses on the networks themselves: we detected emotion-driven feedback loops that influence eating, and we simulated the impact of eating on subsequent emotional state.

Based on the hypothetical negative feedback loops noted earlier, we hypothesized that in AN-R, AN-BP, and BN, eating and binges would predict negative emotions (post-eating dysphoria), and negative emotions would predict reduced eating (restriction). Simultaneously, in AN-BP, BN, and BED, we predicted that negative emotions would predict increased eating, binges, or desire to eat, while in AN-R and AN-BP, *positive* emotions would predict increased eating or desire to eat (emotional eating). As a central exploratory question, we also looked at which specific negative emotions predict binges vs. restriction.

## Methods

### Participants

The final sample comprised 191 women (see Table 1 for details), differentiated into five different groups (HC, AN-R, AN-BP, BN, or BED). Two structured clinical interviews (Structural Clinical Interview for DSM-IV, adapted to DSM-5 criteria; [67], Eating Disorder Examination; [35]) confirmed that the participants met their respective DSM-5 diagnostic criteria.^1^ Participants were excluded from analysis if they were diagnosed with an atypical, other specified, or not otherwise specified ED, if there was no information on their clinical status, if they responded to less than 50% of EMA signals, if they always reported eating 0 calories, or if their demographic data was missing. All participants provided written informed consent and the study was approved by the local ethics committee of Paris-Lodron University Salzburg and the medical ethics committee of Ludwig-Maximilian University Munich.

**Table 1:** Sample characteristics, compared with one-way ANOVAs

Group	*N*	*N* from inpatient waitlist	Age (*SD*)	BMI (*SD*)	% EMA Compliance (*SD*)	Mean *N* binges (*SD*)	% of signals with a meal
HC	57	0	24.23 (8.46)	20.72* (1.52)	83.7 (17.1)	0.17 (0.49)	0.64 (0.12)
AN-R	29	28	26.31 (12.91)	15.75 (2.11)	77.51 (15.12)	0.47 (1.34)	0.64 (0.16)
AN-BP	26	26	25.69 (11.41)	16.42 (1.43)	82.53 (12.35)	4.54 (7.48)	0.54 (0.19)
BN	42	33	28.62 (9.7)	22.98* (2.88)	79.27 (14.84)	4.16 (3.63)	0.57 (0.15)
BED	37	11	36.78* (11.83)	30.59* (7.34)	81.7 (10.85)	2.62 (2.93)	0.64 (0.15)
*F* (4, 186)			8.71	86.3	1.12	12.7	3.4
*p*			< 0.001	< 0.001	0.347	< 0.001	0.01

HC, Healthy controls; AN-R, Anorexia nervosa, restrictive subtype; AN-BP, Anorexia nervosa, binge-purge subtype; BN, Bulimia nervosa; BED, Binge-eating disorder; SD, Standard deviation; BMI, Body mass index; EMA, Ecological momentary assessment

^*^Significant difference from all other groups according to Benjamini-Hochberg-corrected post-hoc *t*-tests

Groups significantly differed in body mass index (BMI), with the AN groups exhibiting the lowest and BED the highest BMI, while the two AN subgroups did not significantly differ from each other (see Table 1). Additionally, those with BED were older than the other groups. Most BED participants were recruited from the general population with flyers, social media posts, newspaper articles, and word-of-mouth, whereas most other participants with an ED were recruited from a waitlist for inpatient treatment. HCs and patients with AN-R reported almost no binges, unlike patients with AN-BP, BN, and BED, validating the diagnostic categories.

### EMA assessment

We administered EMA prompts on the basis of signal-contingent sampling, with six prompts at 9 a.m., 11:30 a.m., 2 p.m., 4:30 p.m., 7 p.m., and 9:30 p.m. Diary entries could be delayed if safety was a concern (e.g., while driving) or when there was no possibility to reply, up to 1 h after the prompt. At each of the six prompts, participants completed questions about their current stress level, emotions, and eating behavior. Momentary emotions were assessed by asking individuals “How do you feel right now?” followed by a list of 7 negative items (i.e., irritated, worried, bored, depressed, tense, nervous/stressed, dissatisfied with self) and 4 positive items (i.e., happy, excited, laid-back, calm) which were presented in random order and to which participants responded by clicking on horizontal rating sliders ranging from 0 (not at all) to 100 (very much). The emotion items were derived from the *Positive and Negative Affect Schedule* [65]. Not all emotions were ultimately analyzed, with some excluded and others merged (see Data Analysis section). Additionally, participants were asked about their momentary hunger (“How hungry are you right now?”) and food craving (“How strong is your desire to eat something tasty right now?”), which were also answered with horizontal rating sliders ranging from 0 (not at all) to 100 (very much).

Participants were instructed to treat occurrences of eating as separate eating occasions if, between moments of food consumption, at least 30 min had passed or if participants had changed location. Thus, eating occasions included snacking, and a main meal was counted as 2 eating occasions if part of the meal had been eaten in one room and the rest in another room. When participants indicated that they had eaten something since the last signal, specific questions for the separate eating occasions followed. Participants answered how many calories each eating occasion contained. As participants were allowed to complete the eating protocol for up to four eating occasions at every prompt, calories were summed per prompt. Participants reported only a single eating occasion 90.25% of the time, followed by 2 eating occasions at 9.24%. Binges were recorded in two ways: (1) participants could indicate they had binged immediately after their binge through the app; and (2) whenever participants reported eating during a regular EMA signal, were also asked if they had experienced binges that they had not reported yet since the last signal. The number of binges reported in realtime and during an EMA signal were summed per signal.

### Procedure

This study was part of a larger project on the momentary dynamics of emotion and eating behavior in patients with EDs and weight-related problems as compared to healthy controls. Hence, the sample of the original study was broader and other self-report [48, 54] and experimental data [21, 59] were collected and published as well; for a full list of published papers based on this sample, see the Appendix.

Participants completed several questionnaires via an online survey platform (including BMI reports). They were then diagnosed via telephone call using the diagnostic interviews noted above, by authors AKA and JR as well as by master students trained by them. During the telephone meeting, they also discussed the installation and usage of the customized smartphone EMA app, as well as the definition of an objective binge. After one practice day, participants completed eight days of EMA assessment, while compliance was monitored by research staff. At the end, participants received another battery of online questionnaires and were remunerated for their participation. For the EMA reported here, participants received € 25–30 depending on their compliance, and they received individualized feedback that was based on their EMA and questionnaire data.

#### Selection and transformation of variables under study

The calorie variable was transformed with formula *x*’ = log(*x* + 1) to enable its use as a linear predictor without undue influence of extreme outliers.^2^ The variables on calm and laid-back emotions were averaged together to create a relaxed emotional state variable, due to a multilevel correlation of *r* (7410) = 0.67 and conceptual overlap; likewise, the variables on stressed/nervous and tense emotions were averaged together into a single variable due to a multilevel correlation of *r* (7410) = 0.59 and conceptual overlap. The excited emotion variable was excluded from analyses entirely due to a multilevel correlation of *r* (7410) = 0.57 and conceptual overlap with happy emotion, model identifiability issues given the large number of predictors, and the lack of a straightforward label for a variable that combines happy and excited mood.

Next, to fulfill the VAR model assumption of stationarity [24], all network variables were temporally detrended, as is common in studies employing VAR (e.g., [46]). Linear temporal trends were removed by predicting each variable with study day (0–7) and measurement moment in the day (0–5); the residuals of this regression were used as variables in the network analysis. This was done separately for each participant. All pre-processing and analysis scripts are available at https://osf.io/sn5wk/.

### Data analysis

#### Temporal network estimation

We estimated temporal networks for each ED group based on modified multilevel VAR models [24]. Like in regular VAR models, we estimated temporal relationships between variables by predicting each variable with all variables from a timepoint directly preceding the predicted value. These predictions were performed with multilevel models with all variables included in the same model as fixed and random effects clustered within participant. The standardized beta coefficients served as edge weights and only significant predictors (*p* < 0.05) were retained as edges. Adaptation of the original VAR model was needed given a discrepancy between which span of time the variables referred to: all emotion, hunger, and craving items referred to the present, while the calorie intake item referred to the past 2.5 h. Calorie intake and binges recorded at t1 were thus modelled as if they occurred between t0 and t1. Since we thus assume that some predictors do not occur at the same time as other predictors, we do not report “contemporaneous” networks from the VAR literature. A full description of this VAR model for asynchronous variables is given in the Appendix. All analyses were performed in R [52], and networks were visualized using the qgraph R package [23]. We only analyzed non-missing EMA prompts for which the previous prompt was also not missing; missing prompts were handled through multilevel estimation (i.e., without imputation, and implicitly assuming missing-at-random).

#### Edge comparisons

Edges were compared between networks using parametric tests based on the beta coefficients and standard errors of the original multilevel models, as suggested by Haslbeck et al. [30]. Given this led to 378 tests, we corrected the *p*-values using Benjamini–Hochberg false discovery rate correction.

#### Network robustness estimation

We tested the robustness of the edges in the temporal network through resampling. To do so, we sub-sampled 75% of the participants 500 times, and computed networks from these sub-samples.^3^ We then counted how often each edge was significant in these resampled networks, as an indicator of how confident we can be that the presence of the edge is supported by the data.

#### Detection of eating-emotion feedback loops

In each network, we detected hypothetical positive and negative feedback loops from an eating-related node to affect-related nodes and back to the same eating-related node (thus, ignoring emotion-emotion and eating-eating loops). This was performed using the R package igraph [19]. We subsequently computed the product of all edge weights per loop to determine whether it is a positive or negative feedback loop. Only loops including up to 5 nodes were considered, as longer loops were likely to lack the strength to be practically relevant.

#### Simulation of future states

We used the temporal network models to predict how a high value in an individual eating-related node (3 *SD*s above the mean) affects values in all nodes at consecutive future timepoints. To do so, we recursively predicted the values of all nodes for 8 future timepoints by multiplying the current values in each node with the outgoing edges of those nodes to determine the value in the destination node at the next timepoint. This yielded simulations of future states for each node, which were characterized by initial peaks that always returned to zero eventually. From these, we computed the simulated trajetory of overall emotional state by summing the simulated values of all emotion variables, which we keyed such that higher values imply a more positive or less negative emotional state.

## Results

In this section, we first report temporal networks per group, and then analyses performed on these networks: these include tests of similarity between the different networks; detection of different feedback loops between eating and emotion; and simulations depicting how eating, craving, and hunger would affect emotions at multiple later timepoints.

### Temporal networks

**Fig. 1:**
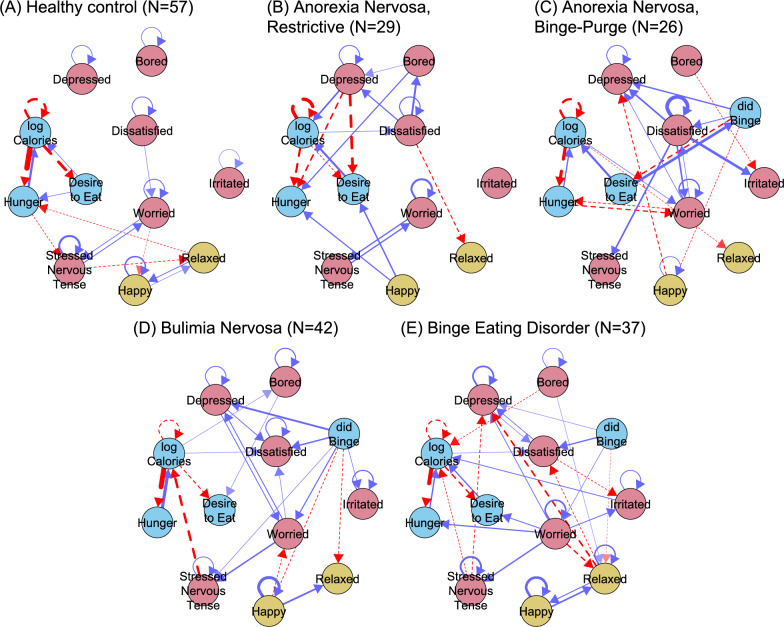
Temporal Networks of the Different Eating Disorder Groups Blue/solid lines signify positive relationships while red/dashed lines signify negative relationships. Larger and more saturated arrows represent stronger effects; the edges in all networks are on the same scale, enabling visual comparisons between networks. Node colors indicate variable type: cyan for eating-related, yellow for positive affect, and pink for negative affect

Various multidirectional relationships emerged between emotions and eating variables; these are depicted in Fig. 1 and will be discussed in the following sections. In contrast to HCs, the different ED groups had many more edges between emotions and eating as noted in Table 2, suggesting a stronger interrelation between the two. Samples were too small to compare all edges between networks with sufficient power while correcting for the large number of tests thus performed. We therefore report the corrected and uncorrected edge comparison tests in the Appendix, but caution against interpreting differences between networks given the insufficient power to detect them. Resampling-based significance rates per edge are also reported in the Appendix as a robustness check.

**Table 2:** Edge counts per network

Group	*N* edges	*N* edges between emotions	*N* edges between emotions and eating
Healthy control	22	14	2
Anorexia nervosa, restrictive	20	9	7
Anorexia nervosa, binge-purge	25	12	7
Bulimia nervosa	28	13	11
Binge eating disorder	35	20	10

#### Effects of eating and related states on emotion

Calorie intake predicted more negative emotion and sometimes less positive emotion in all ED groups, but not in HCs. The same was true for binges in AN-BP, BN, and BED, with particularly strong subsequent negative affect predicted in BN. Hunger predicted reduced worry in AN-BP but predicted no changes in emotion in other ED groups. Hunger also predicted reduced stress/nervousness in the HC group; this effect is likely spurious, as both variables are highly skewed in this sample due to overall low hunger and low negative affect.

#### Effects of emotion on eating and related states

In HCs, only a lack of relaxation predicted hunger, and no other emotions predicted eating-related variables; this contrasted strongly with the ED groups. In AN-R, a strong positive emotional eating pattern was evident, with happiness and a lack of depressed emotion predicting desire to eat and hunger, though dampened by depressed emotion also predicting calorie intake. In AN-BP, worry predicted reduced hunger, while in BED, worry predicted *increased* hunger and desire to eat. In BN and BED, calorie intake was predicted to be lower after stress/nervousness, and in BED also after boredom; however, more calorie intake was predicted in BED after irritation. Binges were not predicted by negative emotion in any ED.

#### Effects among eating and related states, and among emotions

Among eating-related variables, it stands out that in AN-R, hunger did not predict calorie intake, while in BN, desire to eat did not predict calorie intake. Among emotions, it stands out that the AN-R network had relatively less edges among emotion nodes compared to all other ED groups and HCs, suggesting a reduced persistence and spread of emotional states, as noted in Table 2; on the other end of the spectrum, the BED network had the most edges interconnecting different emotions.

### Feedback loop analysis

**Fig. 2:**
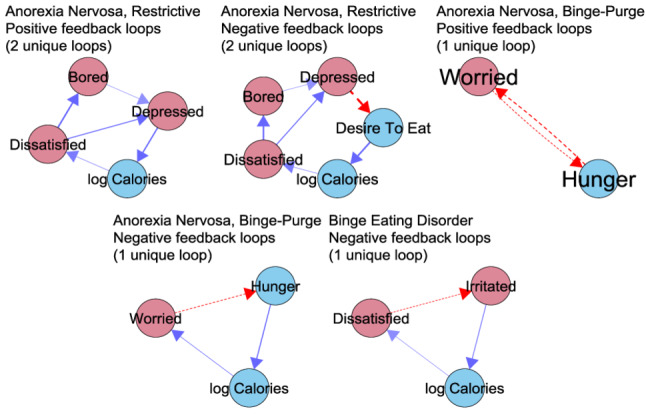
Feedback Loops per Disorder-Specific Network

For each disorder-specific network, the detected emotion-eating feedback loops are reported in Fig. 2; in this section we will illustrate the loops with examples. We do not interpret the 2 feedback loops in the HC network as they hinge on a likely spurious edge (see previous section).

AN-R showed 2 positive and 2 negative feedback loops between eating and emotions. Both followed similar pathways and exerted contradictory influences on calorie intake; consequently, they may cancel out. AN-BP showed 1 negative eating-emotion-eating feedback loop (“eating makes me worry, so I feel less hungry and eat less afterward”), and 1 positive hunger-emotion-hunger feedback loop (“when I don’t feel hungry, I am worried with myself; that worry makes me even less hungry”). BN showed no feedback loops. BED showed 1 negative feedback loop that reduces calorie intake after eating, by indirectly reducing negative emotions that would otherwise predict drive eating: calorie intake predicts dissatisfaction, which predicts reduced irritation, and thereby reduced subsequent calorie intake (“eating makes me feel dissatisfied with myself, but also less irritated; so afterwards I feel less of a drive to eat”).

### Duration of negative affect episodes predicted by elevations in eating-related variables

**Fig. 3:**
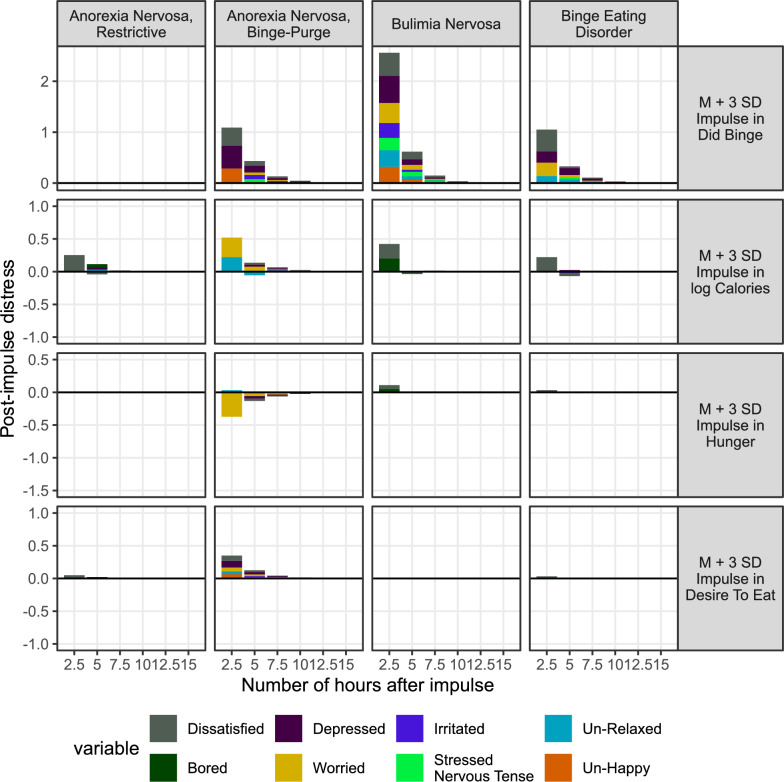
Simulated Trajectory of Emotional Distress Following an Impulse in an Eating-Related Variable

We next report on the simulations of how variables in ED networks would influence each other over time, after an elevation (“impulse”) in an eating-related variable. The results are displayed in Fig. 3. All EDs involving binges reacted with long-lasting (10 h) negative affect to an impulse in binge eating, but in BN the negative affect was especially extreme and spread out across many emotions. All EDs reacted with negative affect to an impulse in calorie intake too; (1) in AN-BP this lasted especially long, and (2) in BED it led to the emergence of positive affect following an initial negative peak. An impulse in hunger led to long-lasting (7.5 h) positive affect in AN-BP, but to minor negative affect in BN and BED due to subsequent eating. Lastly, an impulse in desire-to-eat led to negative affect in AN-R (via calorie intake), in AN-BP (via calorie intake and binging), and in BED (via calorie intake and directly through worry).

## Discussion

This study was the first to investigate bidirectional relationships between emotions and eating behavior using prospective, EMA-based data across the spectrum of EDs. It was also among the first to employ feedback loop detection and future state simulation in this literature.

### Anorexia and bulimia nervosa show restriction after negative affect

We observed a pattern of restriction following negative affect in AN and BN. Patients with AN-R showed positive emotional eating, with happiness and a lack of depression predicting higher desire to eat and hunger, replicating past analyses performed on different parts of the current dataset, which reported elevated self-reported positive emotional eating in AN-R [48, 54]. This positive emotional eating tendency also aligns with what is observed in the general non-clinical population [5]. The flipside of this finding is that individuals with AN-R may eat less when not feeling positive. In AN-BP, worry predicted less hunger, suggesting an indirect pathway towards restriction in response to negative affect in this sample. Evidence for emotional restriction was also present in BN and BED, where stressed/nervous/tense emotion predicted a reduction in food intake. Our findings support the idea articulated in ED models that individuals with EDs restrict their food intake in response to a negative emotional state [26, 58], a finding also confirmed by other empirical studies [12, 22, 25, 54, 59]. These findings complicate the notion that EDs in general are characterized by negative emotional eating, as the positive emotional eating in AN-R stands out as a clear contradiction, and the negative emotional restriction across disorders hints to the idea that at least some forms of negative affect can precipitate restriction rather than eating (in particular, stressed/nervous/tense emotion).

### Binge eating disorder shows negative emotional eating

Besides negative emotional restriction, we also observed signs of negative emotional eating in some of the EDs. In AN-R, boredom predicted hunger, but hunger did not predict eating, while in BN, boredom predicted desire to eat, which did not predict eating; it thus remains unclear whether this represents bored *eating* (termed “boredom-snacking” by [7, 37]). In AN-BP and BN there was an apparent absence of negative or positive emotional eating involving other emotions, contrary to hypotheses. Lastly, patients with BED displayed negative emotional eating through two pathways: worry predicted hunger and desire to eat, and thereby food intake; and irritation predicted food intake directly. These findings echo previous studies that likewise found that anger precedes binges in BED particularly often, though in the current study irritation predicted regular eating and not binges [6, 71]. Interestingly, the current findings highlight that negative emotional eating in BED may at least partially be mediated by desire to eat and changes in the experience of hunger.

One finding across EDs is that binges were not predicted by negative emotion. This is contrary to the body of literature on this topic, and we therefore do not believe that our finding truly implies binges do not follow negative affect; rather, these results suggest that a binge may not be so easily predicted by negative affect *2.5 h in advance*, and therefore, prediction on smaller timescales (e.g., 15 min intervals, in a microEMA design) may be necessary to reveal these effects [9, 38].

### Binges and regular food intake both predict subsequent dysphoria

In line with the models of AN and BN discussed in the introduction [26, 58], our temporal networks revealed that food intake consistently predicted worse emotional state in all ED groups. This was true for regular calorie intake, but also for binge-eating episodes. The predicted direct impact of both on emotional state was exclusively negative, and there were no predicted concomitant improvements in other emotion variables. The negative impact of binges aligns with studies like the current, that investigated the effect of binges on affect at a single timepoint after the binge [2, 29, 34, 66], but not with studies that investigated longer trajectories of affect following a binge [10, 56, 69]. Simulations of future states revealed that the post-binge spike in negative affect could last up to 10 h in AN-BP, BN, and BED, while in AN-BP, such long-lasting (but less extreme) negative feelings were also predicted for regular calorie intake. BN showed the most severe changes in mood following a binge, with predicted increases in many different negative emotions and reductions in several positive ones. The most commonly increased emotion following regular calorie intake was self-dissatisfaction (AN-R, BN, BED) and the most commonly increased emotions following a binge were depression and self-dissatisfaction (AN-BP, BN, BED); this contrasts with the much more prominent role that stressed/nervous/tense emotion had in predicting changes in eating and related variables. All in all, these results highlight that not all negative emotions operate the same way within or across diagnoses.

Even though post-eating dysphoria has been noted before in AN [22, 27], as has post-binge dysphoria in BN [29, 34], the current study robustly finds both phenomena in all EDs. The implications for this finding are clinically significant, as patients may avoid eating full meals purely to avoid the disruption in emotional stability that would otherwise follow, with restriction thus serving as an emotion regulation mechanism.

### Feedback loops between emotions and eating suggest a pattern of post-meal restriction mediated by negative affect

The networks of AN-R, AN-BP, and BED also contained the hypothesized feedback loops wherein eating predicted reduced subsequent eating through a pathway mediated by negative affect. This supports the idea that restriction in these groups is driven by negative affect, which is itself partially driven by food intake. Overall, though there were differences between EDs in the trajectory of eating to affect and back to eating, and though we found no feedback loops for BN, it remains to future research to confirm that these are genuine differences between disorders, as our sample sizes did not offer the statistical power to perform robust comparisons between groups. The same applies to the question of why certain edges exist in one network but not in another—though these may represent real differences between ED groups, we lacked the statistical power to make edge magnitude comparisons, thereby leaving the possibility that some differences between networks may merely be a consequence of low sample size.

### Hunger may perpetuate itself through a positive effect on mood in binge-purge anorexia nervosa

In AN-BP, there was evidence for a positive feedback loop between worry and a lack of hunger: a lack of hunger predicted increases in worry, which in turn predicted even less hunger, in a pattern reminiscent of the classic cognitive-behavioral model of panic disorder [18], wherein physical sensations caused by negative affect likewise cause more negative affect. This is in line with earlier findings that individuals with EDs interpret hunger as a sign of self-control, and conversely, they may interpret a lack of hunger as a loss of self-control [20]. This phenomenon may explain part of the disease process in this disorder, as patients may seek to avoid entering into these prolonged distress spirals by avoiding experiences that reduce hunger (i.e., eating). Further research is needed to study this hypothesis and whether it occurs in other EDs as well.

### Implications for clinical practice and research

Our results have important clinical implications. As treatment foci, they highlight post-eating dysphoria, a panic-like cycle between lack of hunger and elevated worry, and negative affect as a mediator between eating and subsequent restriction. Post-eating dysphoria may be targeted with self-compassion interventions, which were found to be effective in EDs in a handful of studies [33, 36].

Our findings also highlight potentially disorder-specific issues. In AN-BP, we observed elevated distress compared to other EDs (see Appendix), which was also predicted to last longer than in other disorders when triggered by a meal. This indicates an important role for emotion regulation, which was found to improve during treatment, with bigger improvements predicting greater reductions in ED pathology [55]. The fact that patients with AN-BP reported the least eating occasions and the most binges in the sample (Table 1) also speaks to the idea that their eating behavior was the most pathological among the tested groups, giving the impression of an especially severely affected patient group. In BED, we observed clear indicators of emotional eating; this issue could likewise be targeted with emotion regulation trainings, which were previously found to reduce binges in this population [11]. Lastly, if the positive emotional eating in AN-R is replicated, it could serve as a novel pathway to promote food intake in this highly vulnerable population.

Our findings highlight that hunger and desire to eat are somewhat separate concepts that behave in different ways and thus should not be treated as one and the same in EMA research, even if they normally correlate strongly: their relationships with eating and emotion were different from each other across networks. For example, hunger did not predict eating in AN-R, but desire to eat did, highlighting how a more interoceptive signal like hunger is processed differently in this ED [51]. Additionally, though both hunger and desire to eat are antecedents of eating, they had different meanings to patients: in AN-BP hunger predicted reductions in worry, whereas desire to eat did not; while in BED, desire to eat predicted worry, while hunger did not.

The methods of this study also bear relevance for clinical practice and research. All analyses we performed on the networks are also applicable to directed personalized patient networks, for example created through self-report via the L-PECAN method [15] or with longitudinal EMA data and VAR networks. Thus, simulation of future states and feedback loop detection could serve as further tools in the toolbox of the network-informed therapist, alongside traditional centrality indices (of which the utility is questioned; [14]). Likewise, our novel VAR model for asynchronous variables expands the range of symptoms and phenomena that VAR modelling can be applied to.

### Limitations and future directions

The following limitations to generalizability should be acknowledged. The analyzed sample comprised women only. While symptom networks of women and men with different EDs were found to be similar in past research [50], further research is needed to ascertain whether our findings also apply to men.

Most of our participants with AN-R, AN-BP, and BN were recruited from a waitlist for inpatient treatment, and their analyzed behavior may thus reflect some aspect of the treatment they were undergoing, including supervised group meals rather than fully voluntary food intake. We were not able to model differences in meal context since this variable was not measured in the current study. This limits our ability to generalize the data of patients with AN-R, AN-BP, and BN to their daily life outside of treatment. This difference in treatment status also limits their comparability of their data to that of patients with BED, who were predominantly recruited from the general population and thus dealt with generally less severe psychopathology.

Sample sizes were insufficient to enable comparisons between networks while controlling for multiple testing. Hence, we refrained from comparing the networks, and this is left to future research. We advise readers against drawing strong conclusions from the presence of an edge in one network while it is absent in another, as this may also have been a result of low statistical power.

Relatedly, we observed some network features that are highly implausible: depressed emotion, while predicting a lack of desire to eat, also predicted calorie intake in AN-R; and hunger predicted a reduction in stressed/nervous/tense emotion in HCs. The former edge was not present when tested without controlling for the other predictors, so it may be a product of collinearity with the other predictors; LASSO-based predictor selection, as implemented in graphical VAR models, could present a solution to this issue [24], but current implementations lack support for random effects and thus nested data like in this study.

A further limitation is that calorie consumption and binges were fully self-reported. Nevertheless, on average, self-reported binges were associated with a much larger amount of calories consumed, which supports the validity of the binge node for the majority of the sample; this is also supported by the fact that participants were instructed about the definition of an objective binge. The amount of calories consumed was also self-reported, and thus was highly dependent on participants’ ability to judge how many calories were in the food they ate. This latter issue may not be as problematic as appears on first sight, since the subjective aspect of food intake is far more important in the interplay of eating and emotions, but it does mean no conclusions can be drawn regarding relationships of objective calorie intake with other variables.

Lastly, a general limitation of our study design is that all findings are observational; therefore, we can only interpret the reported findings as temporal predictions under specific assumptions, rather than causal relationships.

### Conclusions

The present exploratory study found that regular calorie intake, as well as binges, predicted a subsequent rise in negative affect in all EDs. At the same time, negative affect predicted restriction in AN-R, AN-BP, and BN. These relationships together formed negative feedback loops in AN-R, AN-BP, and BED, with eating or satiety predicting negative affect and thereby reductions in subsequent eating. Emotional eating, by comparison, appeared less prominent in the current data, with positive emotional eating in AN-R (through predicted elevations in craving and hunger), negative emotional eating in BED, and signs of bored eating in AN-R and BN. Taken together, these results give an indication of how and why restriction may occur on a timescale of hours, and where its interventions may best be targeted.

## Figures and Tables

**Fig. 4:**
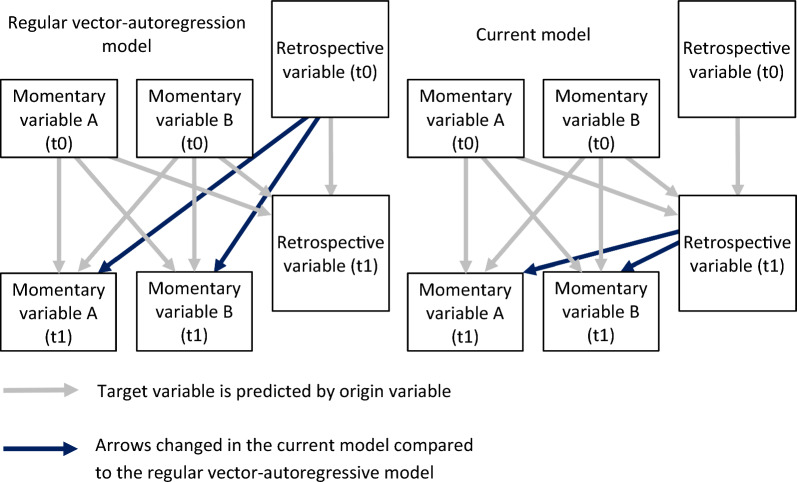
Depiction of the Vector-Autoregression Model for Asynchronous Variables

**Fig. 5:**
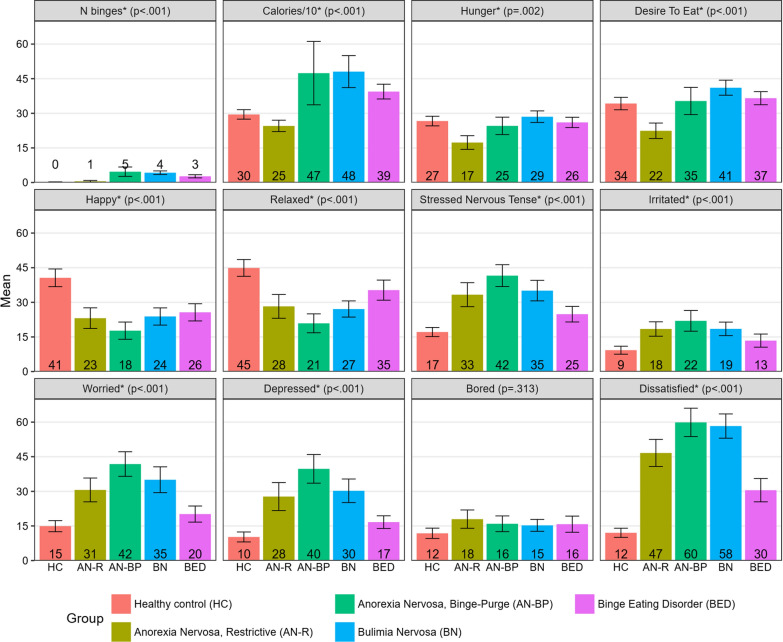
Group Means and 83% Confidence Intervals of Participant Means of all Variables Included in the Networks. Note: Asterisks indicate that group means of the variable significantly differed in a one-way ANOVA. The confidence interval of 83% enables comparison of two means with α = .05. Note that we report raw calorie intake here rather than the transformed variable used in the networks, since this analysis concerned averages rather than individual and sometimes highly outlying observations

**Fig. 6:**
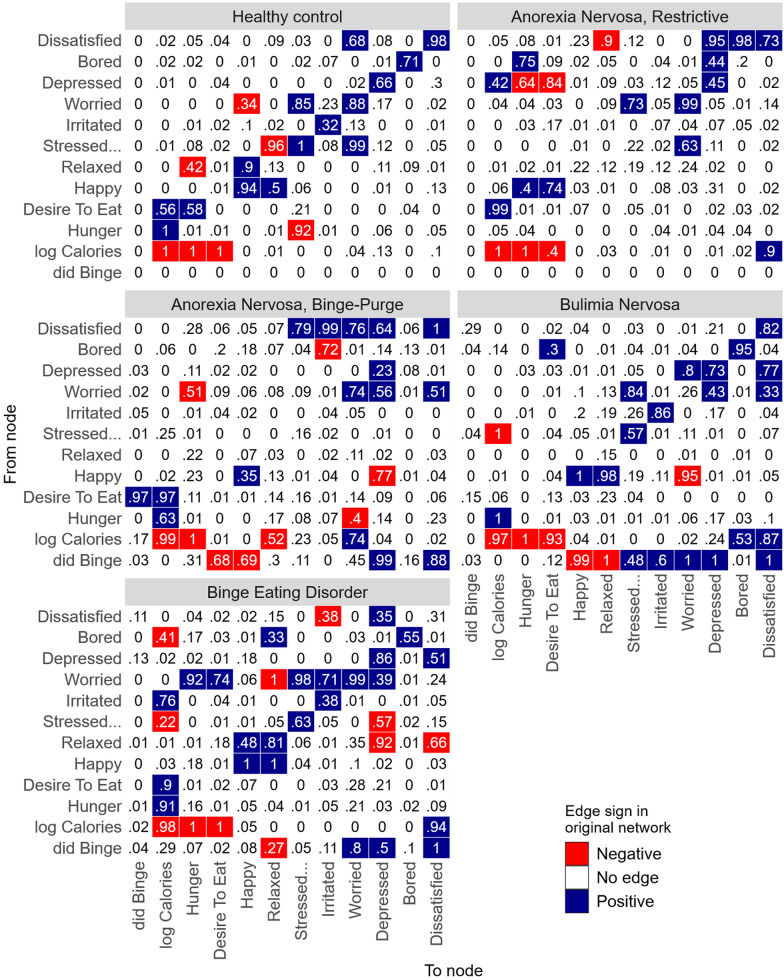
Rate of How Often an Edge was Significant During 500 resamplings. Note: Cells are colored if they represent edges that were included in the original network. A higher value implies a more robust edge. Significance was tested with α = 0.05

**Fig. 7:**
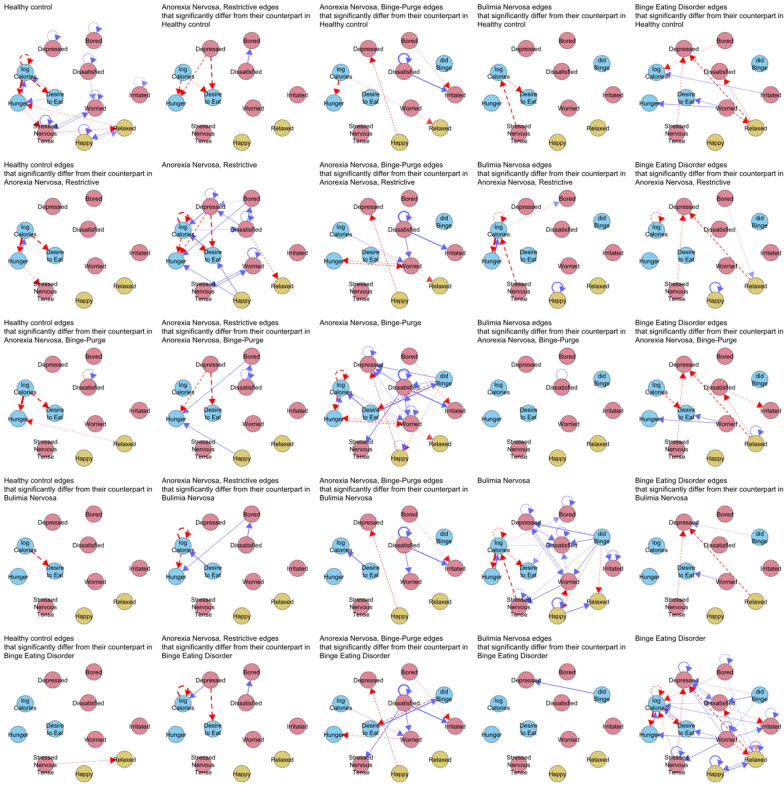
Edge Comparison Tests Between Networks. Note: On the diagonal are the disorder-specific networks. Every off-diagonal network depicts the edges associated with the disorder-specific network in the same column, that significantly differed from the equivalent edges associated with the disorder-specific network in the same row. Blue/solid lines signify positive relationships while red/dashed lines signify negative relationships

## Data Availability

The datasets and analysis scripts supporting the conclusions of this article are available in the Open Science Foundation repository, 10.17605/OSF.IO/SN5WK.

## Appendix

### Previous studies analyzing this same sample

The data used for this manuscript is part of a larger project consisting of several tasks. As such, other papers with a partial sample overlap have been already published. Only one analyzed the same EMA data as this study. In Table 3 is a list with papers from the project that have a partial overlap in data or sample.

**Table 3:** Previous studies based on the same sample as this study

Self-report data	Meule, A., Richard, A., Schnepper, R., Reichenberger, J., Georgii, C., Naab, S., Voderholzer, U., & Blechert, J. (2019). Emotion regulation and emotional eating in anorexia nervosa and bulimia nervosa. *Eating Disorders, 29*(2), 175–191. 10.1080/10640266.2019.1642036
	Reichenberger, J., Schnepper, R., Arend, A.-K., Richard, A., Voderholzer, U., Naab, S., & Blechert, J. (2021). Emotional eating across different eating disorders and the role of body mass, restriction, and binge eating. *International Journal of Eating Disorders, 54.* 10.1002/eat.23477
Computerized tasks	Schnepper, R., Richard, A., Georgii, C., Arend, A., Naab, S., Voderholzer, U., Wilhelm, F. H., & Blechert, J. (2021). Bad mood food? Increased versus decreased food cue reactivity in Anorexia Nervosa and Bulimia Nervosa during negative emotions. *European Eating Disorders Review*, 29(5), 756–769. 10.1002/erv.2849
	Richard, A., Meule, A., Georgii, C., Voderholzer, U., Cuntz, U., Wilhelm, F. H., & Blechert, J. (2019). Associations between interoceptive sensitivity, intuitive eating, and body mass index in patients with anorexia nervosa and normal-weight controls. *European Eating Disorder Review, 27*(5), 571–577. 10.1002/erv.2676
	Georgii, C., Eichin, K. N., Richard, A., Schnepper, R., Naab, S., Voderholzer, U., Treasure, J., & Blechert, J. (2022). I change my mind to get better: Process tracing-based microanalysis of food choice processes reveals differences between Anorexia Nervosa and bulimia nervosa during inpatient treatment. *Appetite, 168*, 105,745. 10.1016/j.appet.2021.105745
	Arend, A.-K., Schnepper, R., Lutz, A.P.C., Eichin, K.N., & Blechert, J. (2022). Prone to food in bad mood—Emotion-potentiated food-cue reactivity in patients with binge-eating disorder. *International Journal of Eating Disorders, 55*(4), 1–6. 10.1002/eat.23683
	Eichin, K. N., Georgii, C., Arend, A.-K., van Dyck, Z., & Blechert, J. (2022). (Mouse cursor)-Tracking food decisions in binge eating disorder reveals preference for high-energy foods and a role of BMI. *Appetite, 170*, 105,890. 10.1016/j.appet.2021.105890
	Eichin, K. N., Arend, A.-K., Reichenberger, J., Voderholzer, U., & Blechert, J. (2026). Food preference and behavioural choice across the eating disorder and body weight spectrum. *Journal of Eating Disorders, 14*(1), 66. 10.1186/s40337-026–01547-4
	Eichin, K. N., Georgii, C., Schnepper, R., Voderholzer, U., & Blechert, J. (2023). Emotional food cue reactivity in Anorexia Nervosa and Bulimia Nervosa: An electroencephalography study. *International Journal of Eating Disorders*, *56*(11), 2096–2106. 10.1002/eat.24028
Ecological momentary assessment	Arend, A.-K., Blechert, J., Yanagida, T., Voderholzer, U., & Reichenberger, J. (2024). Emotional food craving across the eating disorder spectrum: an ecological momentary assessment study. *Eat Weight Disord 29*, 58. https://doi.org/10.1007/s40519-024–01690-4

### Description of the vector-autoregression model for asynchronous variables

The VAR model for asynchronous variables is displayed in Fig. 4. Like in regular VAR models, retrospective calorie intake (t1) was predicted by all variables from the previous EMA signal, since these values precede it in time (t0); but also, each momentary variable (hunger, desire to eat, and all emotions) (t1) was predicted with retrospective calorie intake from the *current* EMA signal (t1) and with all momentary variables from the previous EMA signal (t0), since these values precede the predicted value in time.

### Descriptives of EMA items

We report mean values of each EMA variable per group in Fig. 5. Groups significantly differed on many of the EMA variables, with boredom being the only variable without significant group differences. Binges did not occur in patients with AN-R or healthy controls, validating the diagnostic categories and the binge item. Patients with AN-R reported the lowest calorie consumption, hunger, and desire to eat. Patients with AN-BP reported the lowest degree of relaxation and the highest degree of stress/nervousness/tension, worry, and depressed mood. Among patient groups, those with BED reported the highest levels of relaxation and the lowest levels on many of the negative affect variables, with values close to those of healthy controls; this is likely because many of these participants were recruited from the general population and not from a waitlist for inpatient treatment, in line with lower rates of inpatient treatment for this population. Their relatively lower calorie intake compared to BN may also reflect the lower disorder severity among the BED sample, and it corresponds with the finding that calorie intake in BED is not elevated outside of binge episodes [53].

### Robustness of individual edges

The significance rates per edge are displayed in Fig. 6.

### Testing for significant differences between networks

When correcting all edge comparisons for false discovery rate, only 2 edges significantly differed between networks given these very conservative testing parameters. Desire-to-eat predicted much more subsequent calorie intake in patients with AN-BP than in HCs, *p* = 0.014; and self-dissatisfaction predicted more irritation in AN-BP while predicting less irritation in BED, *p* = 0.027. For exploratory purposes, we report the edges that significantly differed between networks with an uncorrected α level of 0.05 in Fig. 7. These should be interpreted with care given the very high likelihood of chance findings.

## Abbreviations

ED: Eating disorder
AN: Anorexia nervosa
AN-R: Anorexia nervosa, restrictive subtype
AN-BP: Anorexia nervosa, binge-purge subtype
BN: Bulimia nervosa
BED: Binge-eating disorder
HC: Healthy control
VAR: Vector autoregression
EMA: Ecological momentary assessment
BMI: Body mass index
SD: Standard deviation

## Footnotes

1 Since there were no German-language interviews for the evaluation of DSM-V criteria at study start in 2015, these older interviews were adapted to DSM-5 diagnostic criteria, like requiring one instead of two binge-eating episode per week for three months.

2 We also recoded 1 observation of 36,360 cal to 360 cal; the original value was likely an imputation error since the participant reported not consuming an extreme amount of food on another question at the same timepoint.

3 We did not sample 100% of the original sample size with replacement, as this led to convergence issues with the model.
